# Management of Ejaculatory Duct Obstruction by Seminal Vesiculoscopy: Case Report and Literature Review

**DOI:** 10.5935/1518-0557.20190075

**Published:** 2020

**Authors:** Filipe Tenorio Lira Neto, Phil Vu Bach, Eduardo de P. Miranda, Sergio Luis da Silva Calisto, Guilherme Maia Tavares da Silva, Dimas Lemos Antunes, Philip S Li

**Affiliations:** 1 Andros Recife, Brazil; 2 Universidade Federal de Pernambuco, Brazil; 3 Instituto de Medicina Integral Prof. Fernando Figueira, Brazil; 4 University of Alberta, Canada; 5 Universidade Federal do Ceará, Brazil; 6 Centro Universitário Maurício de Nassau, Brazil; 7 Weill Cornell Medicine, USA

**Keywords:** seminal vesicle, male infertility, hematospermia, endoscopy

## Abstract

Ejaculatory duct obstruction is a rare condition identified in up to 5% of infertile men. Patients with ejaculatory duct obstruction can present with aspermia, azoospermia or oligoasthenospermia, painful ejaculation, hematospermia, prostatic pain, or male infertility. Semen analysis, transrectal ultrasonography, pelvic computerized tomography and magnetic resonance imaging are often used in the diagnostic work up, but with limited accuracy. While transurethral resection of the ejaculatory ducts has good efficacy for distal duct obstruction, results for proximal obstruction are less impressive, and it might cause severe complications, such as rectal injury and urinary incontinence. Recently, the use of high quality endourological devices and an improved understanding of ejaculatory ducts anatomy gleaned through the use of sophisticated imaging tools have led to the development of novel minimally invasive treatment options for this condition. The present study aims to report an index case of ejaculatory ducts obstruction managed with seminal vesiculoscopy, and review the current literature regarding this topic.

## INTRODUCTION

Ejaculatory duct obstruction (EDO) is a rare condition present in up to 5% of infertile men. It may be caused by several pathologies such as ejaculatory duct (ED) malformations, midline prostatic cysts, fibroses due to prostatitis or seminal vesiculitis, seminal vesicle (SV) stones, or scarring after endoscopic manipulation (Kang *et al*., 2016; Wang *et al.*, 2012). Patients with EDO can present with aspermia, azoospermia or oligoasthenospermia, painful ejaculation, hematospermia, prostatic pain, or male infertility. Semen analysis (SA), transrectal ultrasonography (TRUS), pelvic computerized tomography (CT) and magnetic resonance imaging (MRI) are often used in the diagnostic work up of EDO, though have limited accuracy in identifying the etiology of EDO (Han *et al*., 2009; 2013).

Despite being known for decades, EDO is considered a problem of difficult management due to the complex anatomic relations of the ejaculatory ducts (ED). While transurethral resection of the ejaculatory ducts (TURED) has good efficacy for distal duct obstruction, results for proximal duct obstruction are less impressive, and it might cause severe complications such as rectal injury and urinary incontinence (Kang *et al*., 2016; Wang *et al.*, 2012; Han *et al*., 2013). Recently, the use of high quality endourological devices and an improved understanding of ED anatomy gleaned through the use of sophisticated imaging tools have led to the development of novel minimally invasive therapeutic options for EDO. The present study aims to report an index case of EDO treatment with seminal vesiculoscopy (SVC), describe the technique of SVC, and review the current literature. This report was approved by the Research Ethics Committee of our institution (approval number 2.057.579).

## CASE REPORT

The patient was a 44-year-old healthy male, presenting with a three-year history of persistent hypospermia and recurrent episodes of hematospermia. He also had primary infertility, being unable to conceive with his 42-year-old wife, despite regular unprotected intercourse in the past 10 years. He was treated empirically with several antibiotic regimens and phytotherapeutic supplements without any improvement in symptoms. His past medical history was significant for obesity, having been submitted to bariatric surgery 3 years previously, and untreated bilateral varicoceles. His physical exam revealed bilateral grade II varicoceles, a 15 mL right testis, a 12 mL left testis, and bilateral normal epididymis, and vasa. Digital rectal exam was unremarkable, with non-tender prostate and non-palpable SVs.

The patient’s initial standard SA showed a decreased semen volume (0.5 mL, reference: >1.5 ml), hematospermia (3,700 red blood cells, reference: none), decreased total sperm count (300,000 sperm, reference: >39 million), decreased progressive motility (20%, reference: 32%), and borderline morphology using the strict Kruger criteria (4%, reference: >4%). A post-ejaculate urine analysis showed 300,000 immotile sperm in the pellet and a negative semen culture. Serum sexual hormone levels revealed normal serum total testosterone (616 ng/dL) and estradiol (31.2 pg/mL) levels, but slightly increased serum follicle-stimulating hormone (9.31 mIU/mL) and luteinizing hormone (11.8 mIU/mL) levels. Scrotal color Doppler ultrasonography evidenced bilateral varicocele, with the largest internal spermatic vein measuring 3mm on the left side and 2.5mm on the right side.

**Figure 1:**
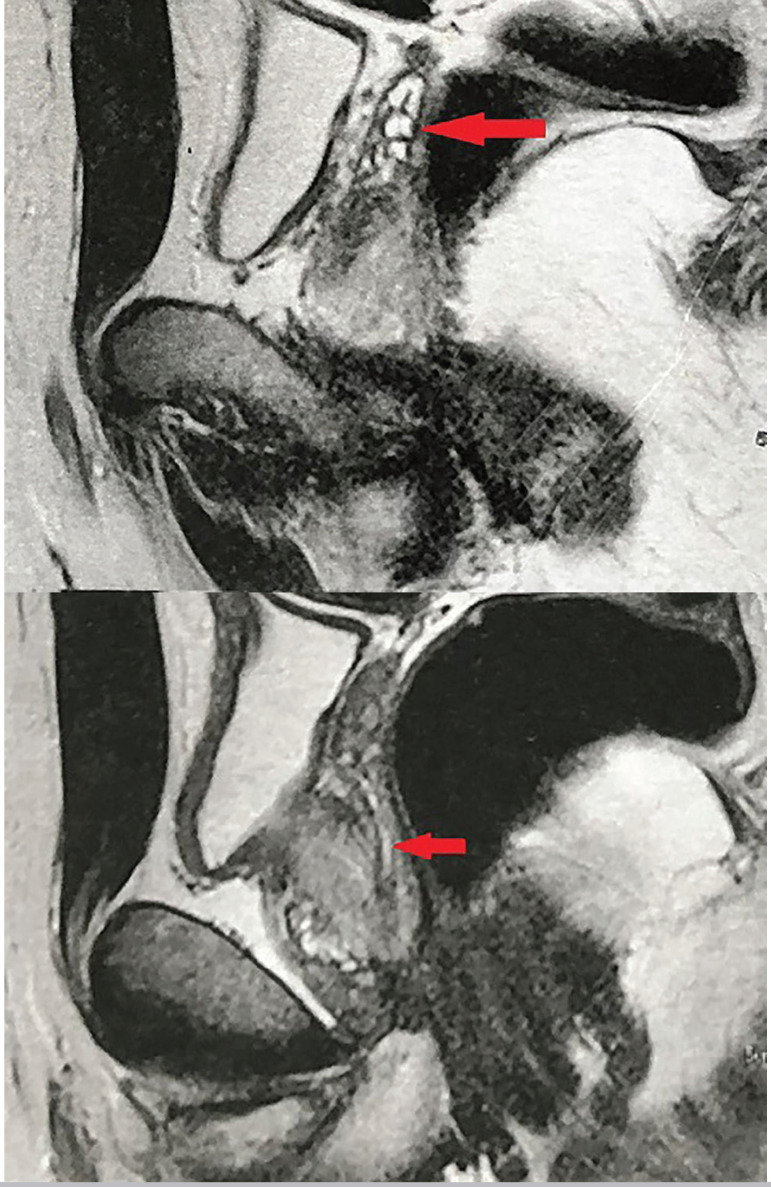
MRI. Dilation of the right SV and ED with no signs of inflammatory, neoplastic or cystic lesions in the prostate and SVs

Since the patient refused to perform TRUS, further diagnostic evaluation was done with contrast-enhanced pelvic MRI, which revealed dilation of the right SV and ED with no signs of inflammatory, neoplastic or cystic lesions in the prostate and SVs (Figure 1).

After the patient was informed of the probable diagnosis of partial EDO, and spermatogenesis impairment secondary to varicoceles and bariatric surgery, the patient agreed to undergo endoscopic SVC aiming to confirm the diagnosis of partial EDO and to improve the hematospermia and his SA parameters.

**Figure 2:**
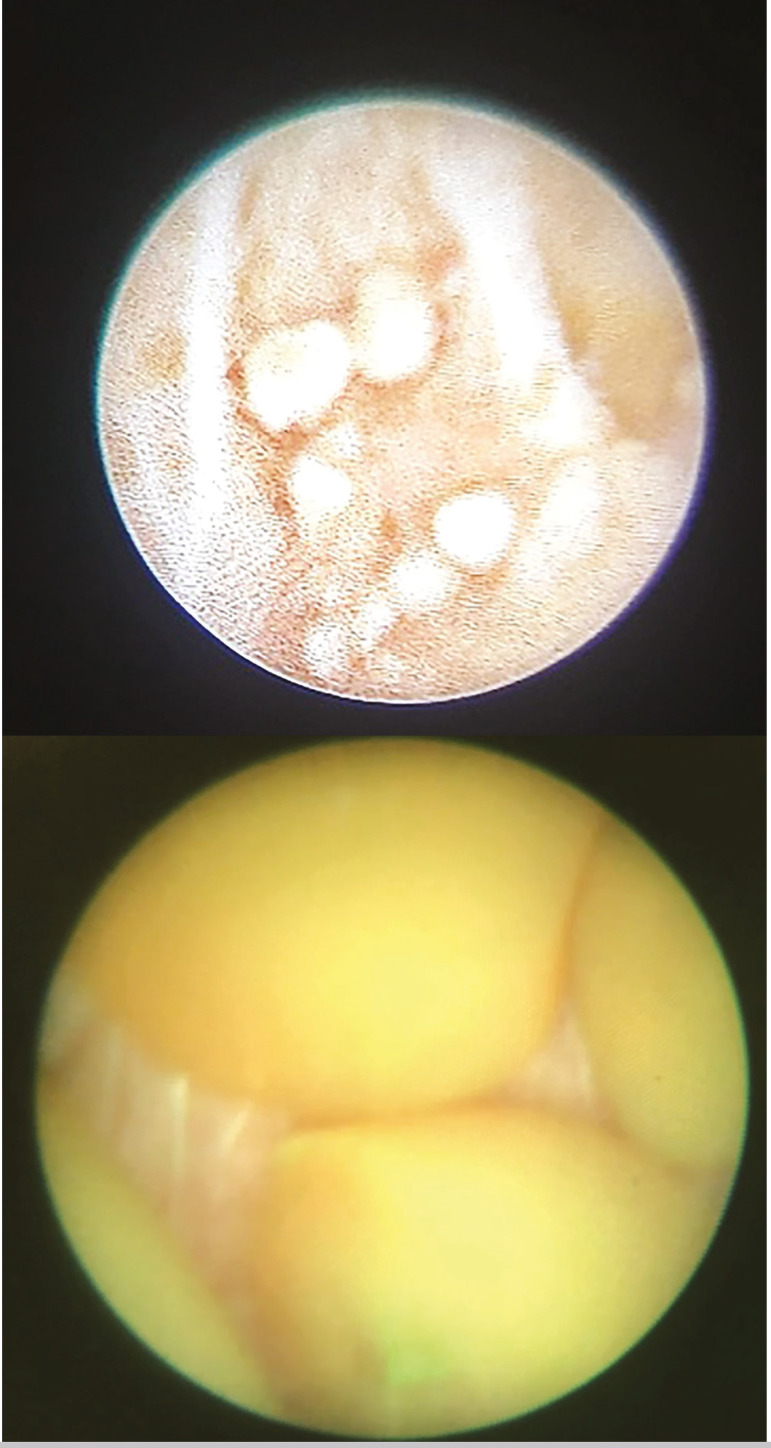
Ureteroscopy right ED. Several smalls stones and amorph material were found

The procedure was performed in lithotomy position under general anesthesia. Ciprofloxacin was given as antibiotic prophylaxis. Urethroscopy was performed using a 6 Fr rigid ureteroscope and the verumontanum was identified. Catheterization of the ED orifices with a hydrophilic guidewire was unsuccessful. Careful inspection of the internal cavity of the prostate utriculum revealed no connection to EDs. Unroofing of the verumontanum with a 26Fr resectoscope using monopolar cut current was then performed, and drainage of dark and haze fluid was observed coming from the right ED. This maneuver allowed the guidewire to be inserted into the right ED and the ureteroscope was progressed in the right seminal vesicle. Intermittent low-pressure irrigation and gentle alternate rotation of the scope was used. Several smalls stones and amorph material were found (Figure 2). Irrigation was used again to flush out all the material and stones. Revision of the right seminal vesicle revealed dilated right ED and absence of residues. Using the right ED as a landmark, the left ED was successfully catheterized, and a milky liquid drained after guidewire insertion and the vesiculoscopy revealed a small amount of amorph material.

The final endoscopic evaluation revealed an unroofed prostatic utriculum and the dilated right and left ED orifices at 2 and 10 o’clock positions, respectively. No significant bleeding was observed. Bladder inspection revealed no abnormalities, and a digital rectal examination was negative for blood. At the end of the procedure, an 18 Fr urethral Foley catheter was left in place overnight. The patient was discharged on the following day and was counseled to resume sexual activity as soon as possible to maintain ED patency.

A SA performed on the 30th postoperative day revealed normal ejaculate volume (2.0 mL), no red blood cells, an increase of the total sperm count to 1.000.000/ejaculate, improved morphology (10%), and unchanged progressive motility (20%). After the procedure, he reported no new episodes of hematospermia, denied any sexual symptoms such erectile or ejaculatory dysfunctions, and noticed a subjective feeling of increased ejaculatory volume. He also denied having pelvic pain or symptoms of prostatitis, seminal vesiculitis or epidydimitis.

## DISCUSSION

*In vivo e*ndoscopic evaluation of the seminal vesicles was first reported by Okubo *et al*. (1998). Yang *at al*. (2002) published the first large study with 37 patients. They performed a transutricular access in men with persistent (>3 months) hematospermia and SV abnormalities on imaging studies. Their technique consisted of catheterization and dilatation of the utricular orifice with guidewire and a 5 Fr open-ended ureteral catheter followed by inspection of the utricular lumen using 6 Fr and 9 Fr rigid ureteroscopes. The EDs were accessed by inserting the ureteroscope in directly into the ED orifices via the utricular lumen. A coagulating electrode was used to open the utricular orifice or the ED when these structures were not immediately visible (Yang *et al*., 2002). The same group published a larger series of 70 patients with persistent hematospermia in 2009 (Han *et al*., 2009). In the subsequent paper, they injected dye antegrade through the vas deferens to confirm the location of the ED orifices and resected the verumontanum to find the orifices in those cases where the verumontanum had been previously damaged. After a mean follow-up of 12.3 months, 55 patients had resolution of their hematospermia while no complications were reported. Hematospermia recurred in 7 cases (Han *et al*., 2009).

Using a similar transutricular ED access technique, Liu at al published a cases series of 72 hematospermic patients managed with SVC (Liu *et al*., 2009). They noticed that the ED orifices inside the utricular lumen were always covered by a transparent membraniform wall. Definitive diagnosis and symptomatic improvement were achieved in 93.1% and 97.2% of the patients, respectively; with no complications after a median follow-up of 21.7 months (Liu *et al*., 2009).

According to Guo *et al.*, the ED can also be catheterized directly from the urethra without entering the utricular lumen (Guo *et al*., 2015). The ED orifices can be found outside the prostate utricle, usually at 5 and 7 o’clock positions and, if the orifices are unclear, the verumontanum can be resected to expose their openings. In their case series of 20 patients, an epidural catheter was used as guide and for flushing saline solution in order to identify the ED openings.

A prospective trial conducted by Xing *et al.* compared the diagnostic yield of TRUS and SVC in 106 patients with persistent hematospermia (Xing *et al*., 2012). Seminal vesiculoscopy could not be performed in 7.5% of the patients because the ED orifices were not identifiable. The individual diagnostic yields of TRUS and SVC were 45.3% and 74.5%, respectively (*p*<0.001); with the overall diagnostic yield rising to 87.7% when the modalities were combined. Calculi (87%) and strictures (79.6%) were the most common findings on SVC. Therapeutic interventions were performed in 83.3% of the patients who underwent SVC, with 97.6% having resolution of their hematospermia. Twenty-three patients (21.7%) developed temporary mild perineal pain that resolved spontaneously in less than 3 months and no serious complications were reported. The authors concluded that combining TRUS and SVC might improve the management of men with persistent hematospermia.

The efficacy of SVC for the treatment of complete EDO was assessed by Wang *et al.* in a series of 21 azoospermic patients with EDO. The procedures were performed using the same transutricular technique described earlier (Wang *et al.*, 2012). One patient required TURED because of failure to identify the ED orifices. Only 2 patients remained azoospermic after 12 months post-surgery, with the mean sperm count rising from 0 to 6.6x10^6^/mL, and the mean semen volume increased from 1.1 mL to 2.8 mL after 3 months. Again, perineal discomfort was present in 7 patients after the procedure, but the pain subsided in all patients after 3 months, and no major complications were reported. The authors noted that a 6 Fr rigid ureteroscope was more effective when performing SVC, and the ED orifices were usually found next to the median line of the verumontanum (Wang *et al.*, 2012).

Han *et al.* reported a case series including 61 men with seminal vesicle disease. Using an F 6/7.5 ureteroscope, SVC was successfully performed in 95% of the cases, with a mean surgical time of 35.6 minutes. Only 2 patients complained of perineal discomfort after the procedure, and 1 patient had recurrence of hematospermia (Han *et al*., 2013). A similar success rate with SVC was demonstrated by Hu et al. In their 38 patient-case series, SVC had a success rate of 92.1%. Interestingly, even the 17 cases with negative findings improved their symptoms after the procedure. The recurrence rate was 11.8%, and 5.2% of the men developed post-operative epididymitis, treated with antibiotics. Another large series of 114 patients with hematospermia and abdominal or perineal pain demonstrated resolution of the hematospermia and pain improvement after SVC in 89% of the cases. There were 2 cases of postoperative epididymitis, 6 cases of postoperative painful ejaculation and no major complications (Liu *et al*., 2014).

In the largest series up to this date, Liao *et al.* reported the outcomes of 305 cases of refractory hematospermia treated with SVC (Liao *et al*., 2019). The procedure was successfully performed in 296 patients, and all 271 treated men who had follow-up experienced resolution of hematospermia. Seven percent of the patients developed recurrent hematospermia, treated with a second procedure. Complications were rare, 5.9 men complained of thinner ejaculation and only one case of epididymitis was reported. No case of perineal pain was observed after the procedure.

The group of Zhang *et al.* described the use of SVC coupled with ultrasonic lithotripter to treat patients with persistent hematospermia. In a retrospective study, 30 patients were divided in two groups, 16 who underwent conventional SVC (group A), and 14 who underwent SVC with ultrasonic lithotripter (group B). Overall, 56% of the men had calculi in the SV and surgical time was shorter in group B (55 *versus* 66 minutes). All the procedures were successful in group B, while 1 patient in group A had the procedure interrupted due to bleeding. There were no recurrences in group B and 2 in group A. In both groups, there were no complications. The authors advocated the use of ultrasonic lithotripter due to its strong and continuous suction, providing a clear surgical field and minimizing the SV pressure (Zhang *et al*., 2017).

Kang *et al.* evaluated the use of SVC for the treatment of symptomatic prostate midline cyst diagnosed by TRUS in 61 patients (Kang *et al*., 2016). The main presenting symptoms were hematospermia (52.4%) and chronic pelvic pain syndrome symptoms (32.7%). Fifty-seven percent of the patients had seminal vesicle dilation (>12 mm) on TRUS, and 28% had calculi found in the midline cyst during SVC. The SVs were successfully accessed in 53 cases. Hematospermia resolved in 90.6% of the cases (with only 1 recurrence) and the prostatitis symptoms improved significantly after the procedure (Kang *et al*., 2016). Surprisingly, SA parameters did not improve in this cohort. Two men developed acute epididymitis and 2 other minor complications were reported.

When a midline prostate cyst is a suspected cause of EDO, unroofing of the cyst using a resectoscope combined with SVC can be used. Cheng *et al.* described a series of 12 infertile men with midline prostate cysts treated with the combined procedure and reported improvements in semen quality in 80% of the men (Cheng *et al*., 2015).

The use of SVC to treat SV cysts was evaluated by Xue *et al*. (2018). The technique includes a transutricular approach and holmium laser was used to create a communication between the cyst and the SV lumen. Twenty men with SV cysts ranging from 32 to 55 mm were treated. Although the procedure was well tolerated, with 8 patients developing self-limited mild hematospermia, symptomatic improvement accessed by NIH-CPSI did not reach statistical significance and no patients were free of cystic lesions on follow-up. 

Seminal vesiculoscopy is a technique that can be used to treat several conditions from the prostate, EDs, and SVs. Evidence is growing in support of its use as an effective alternative to more invasive procedures like TURED, since the procedure uses commonly available urologic equipment such as cystoscopes, rigid 6-9 Fr ureteroscopes, resectoscopes, guidewires, ureteral catheters, and coagulating electrodes. On the other hand, a strong knowledge of the pelvic anatomy is required and the surgeon must be able to recognize small structures and anatomic landmarks while discerning which maneuvers will safely lead to the SVs.

As per described above, the ED orifices can be accessed via two different approaches - the transutricular approach or the direct approach via the urethra at the 5 and 7 o’clock positions of the prostatic utricle. The most commonly described access is the transutricular approach, which involves using a ureteroscope and a guidewire to enter the utricular lumen before catheterizing the ED orifices or puncturing the thin lateral wall that sometimes covers the ED orifices from within the utricular lumen. The second direct approach to the ED orifices is performed by catheterizing the natural ED orifices directly from the urethra, which is more difficult due to the small size of the openings. Finally, if both of the aforementioned approaches fail, one may resect the verumontanum to unroof the EDs that run postero-laterally to it. However, this technique should be used as a last resource since it has the potential to cause complications such as reflux epididymitis, urinary incontinence, and rectal injury.

SVC can be regarded as a safe and effective treatment modality for patients with EDO, hematospermia, and some pelvic pain conditions. The procedure is feasible in most patients and outcomes are excellent, with hematospermia resolution rates ranging from 78% to 98% and recurrence rates as low as 10% (Han *et al*., 2009; Liu *et al*., 2009; Xing *et al*., 2012; Liu *et al*., 2014). Pelvic pain or ejaculation-related pain can also improve after SVC (Kang *et al*., 2016; Liu *et al*., 2014), though up to 30% of the patients may develop perineal pain or discomfort in the postoperative period (Han *et al*., 2009). Although these symptoms are mild and temporary, patients should be informed about this possibility. Postoperative epididymitis seems to be rare and may occur due to urine reflux to the epididymis caused by the destruction of the ED orifice valve mechanism during dilation. High pressure irrigation during the procedure may also cause epididymitis. No other major complications have been described in the literature, which might have been underreported because of insufficient follow-up time. Larger series and longer follow-up are necessary to establish the long-term efficacy and safety of SVC, and to identify the conditions that would benefit at most from this procedure. In addition, studies from different populations are needed to compare results in patients with distinct anatomical variants, and postoperative recommendations should be standardized.

## CONCLUSION

Early reports suggest SVC to be a safe and feasible technique that represents a minimally invasive treatment modality for EDO, persistent hematospermia, and some pelvic pain conditions. It seems to be as effective as TURED, with lower potential complication rates, but further studies are required to clarify long term outcomes and to provide external validation.
